# Patient-specific coronary artery supply territory AHA diagrams

**DOI:** 10.1186/1532-429X-11-S1-P103

**Published:** 2009-01-28

**Authors:** Maurice Termeer, Javier Oliván Bescós, Marcel Breeuwer, Anna Vilanova, Frans Gerritsen, Eduard Gröller, Eike Nagel

**Affiliations:** 1 Vienna University of Technology, Wien (Vienna), Austria; 2 Philips Healthcare, Best, Netherlands; 3 Eindhoven University of Technology, Eindhoven, Netherlands; 4 King's College London, London, UK

**Keywords:** Coronary Artery, Left Ventricle, Voronoi Diagram, Quadrilateral Mesh, Epicardial Surface

## Introduction

The American Heart Association proposed a 17-segment model for the segmentation of the left ventricle together with a mapping from each segment to a supplying coronary artery. This proposal is based on population averages. Several studies have confirmed the inaccuracy of this mapping due to large anatomical variations of the coronary arteries among individuals. Several proposals have been made for a different mapping between the 17 segments and the coronary arteries.

## Purpose

Due to the large variation in coronary anatomy there is a need for a patient-specific assignment of ventricular segments to supplying coronary arteries. We propose to use a segmentation of the coronary arteries and the ventricular epicardium to compute this patient-specific mapping.

## Methods

The three primary coronary arteries (LAD, LCX and RCA) and the left ventricle are segmented in a whole-heart MRI (SSFP) or CT scan of at least 150 slices. For the coronary arteries we employ a semi-automatic vessel tracking algorithm. The left ventricle is segmented using a fully automatic approach. The epicardial surface of the resulting segmentation is represented as a quadrilateral mesh. The centerlines of the coronary arteries are projected on the epicardial surface. A Voronoi diagram of the projected arteries is computed using a geodesic distance metric. The patient-specific coronary supply territories are computed using a modified marching squares algorithm. The examples given here consist of three territories, but our approach is flexible enough to handle any amount of territories.

**Figure 1:**
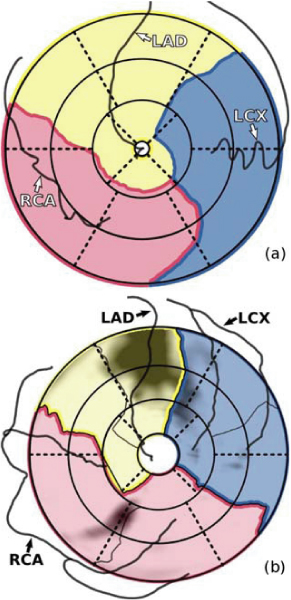
**(a) Bull's eye plot showing patient-specific coronary supply territories**. The dotted lines represent the 17-segment model. (b) Patient-specific coronary supply territories as an overlay on a bull's eye plot of a late enhancement scan.

Both the coronary supply territories and the coronary arteries are projected onto a bull's eye plot using a parameterization of the left ventricle based on cylindrical coordinates, using the cardiac long axis as the primary axis of the cylinder (Figure 1a). The continuous nature of the epicardial surface is preserved in this projection. This means that the bull's eye plot does not consist of rings representing slices, but that the distance to the center is proportional to the distance to the apex. This bull's eye plot can for example be used as an overlay for the analysis of viability (Figure 1b).

## Results

We evaluated our method on image data from five patients. For each patient we produced both a standard 17-segment diagram and a diagram with the projection of the patient-specific coronary supply territories resulting from our approach. In both diagrams a projection of the segmented coronary arteries was shown. We then asked an experienced clinician to judge the correspondence between the coronary arteries and the suggested coronary supply territories for both diagrams. It was judged that our patient-specific coronary supply territories provide a better correlation with the position of the coronary arteries. The clinician expressed a preference to our method as compared to the standard 17-segment model.

The continuous relation between the distance to the center of the bull's eye plot and the distance to the apex caused some confusion with our clinician. Especially in combination with CMR data consisting of relatively few slices this relation should be clarified.

## Conclusion

With our method the relation between coronary arteries and areas supplied by these arteries is better visualized. This will help to better correlate the location of infarcted or ischemic areas to the coronaries that have caused the respective infarction or ischemia.

